# Age-dependent change of coalitionary strategy in male Barbary macaques

**DOI:** 10.5194/pb-4-1-2017

**Published:** 2017-01-26

**Authors:** Eva-Maria Rathke, Andreas Berghänel, Annie Bissonnette, Julia Ostner, Oliver Schülke

**Affiliations:** 1 Department of Behavioral Ecology, Georg-August University Göttingen, Kellnerweg 6, 37077 Göttingen, Germany; 2 Cognitive Ethology Laboratory, German Primate Center, Leibniz Institute for Primate Research, Kellnerweg 4, 37077 Göttingen, Germany; 3 Department of Anthropology, University of New Mexico, MSC01-1040, Anthropology, 1, Albuquerque, NM 87131, USA; 4 Anthropological Institute and Museum, University of Zürich, Winterthurerstrasse 190, 8057 Zürich, Switzerland; 5 Primate Social Evolution Group, German Primate Center, Leibniz Institute for Primate Research, Kellnerweg 4, 37077 Göttingen, Germany

## Abstract

Inter- and intra-specific variation in the propensity to form coalitions has been explained by variation in the availability of suitable partners, distribution of fighting ability, coalition profitability, and costs of coordination. Male coalition formation can be an alternative reproductive strategy to one-on-one competition to maximize male reproductive success. Here we focus on age as a state variable to explain within-group variation in individual propensity to form coalitions against other group members. We specifically test the prediction that males conditionally switch from a solo strategy for achievement of high mating success to a cooperative strategy after reaching post-prime age in male Barbary macaques (*Macaca sylvanus*). We combined new observations with data collected in 2006 and 2008 on the same individuals from one captive group living in semi-natural conditions at Affenberg Salem, Germany, and found that in all years males between 5 and 13 years formed significantly fewer coalitions than males 14 years and older (post-prime). More importantly, we found those males that aged into the post-prime phase to have switched their reproductive strategy and to form significantly more coalitions in 2014 compared to 2008. These first longitudinal data together with earlier cross-sectional analyses in this and other primate species suggest that group-level measures of coalition propensity may be strongly affected by the age composition of groups and that male coalition formation can be a conditional reproductive strategy.

## 1 Introduction

To maximize lifetime reproductive success males often engage in alternative reproductive strategies in order to gain access to females, which is limited by several inter- and intra-individual factors (Setchell, 2008). Access to females is constrained by synchronized female receptivity, direct competition among males, and female choice, all of which affect a male's competitive style within a group (Noë and Sluijter, 1990; Preuschoft and Paul, 2000). In contrast to subordinate males, the highest-ranking males are expected to profit from mate guarding and competing solely due to their own competitive strength (Noë and Sluijter, 1990). Subordinate males can maximize their individual reproductive success by affiliating with females and thereby obtaining access to them or by alternatively pursuing more competitive strategies like coalition formation where two or more males aggressively join against a third party (Bercovitch, 1988; Bissonnette et al., 2011; Noë and Sluijter, 1990; Smuts, 1985). However, there are costs of cooperation, including a less predictable outcome with regard to mating success since just one male can fertilize a female (Krützen et al., 2003; Packer and Pusey, 1982; Smith et al., 2010; Wittig and Boesch, 2003). Still coalitions are widespread in several mammalian species and have been found to effectively lower male reproductive skew in bottlenose dolphins (*Tursiops* sp., Connor et al., 1992), savannah baboons (*Papio cynocephalus*, Noë and Sluijter, 1995), lions (*Panthera leo*, Grinnell, 2002), wild dogs (*Lycaon pictus*, de Villiers et al., 2003), and in several primate species (Alberts, 2012; Dubuc et al., 2011). Coalitions function in various ways to increase male mating success: interrupting a higher-ranking opponent's consortship and to start consorting the female themselves can be a direct way for males to gain access to females (Bercovitch, 1988; Bissonnette et al., 2011). In many cases, however, females are not present during a coalitionary conflict, which suggests that coalition formation can also function as an indirect pre-mating strategy via intimidation of the opponents and thereby influencing the current dominance hierarchy (Bissonnette et al., 2011; Berghänel et al., 2011; Young et al., 2014a). Later mating opportunities could then be improved by previously having threatened the potential opponent (Noë and Sluijter, 1990; van Schaik et al., 2006; Berghänel et al., 2010).

Inter- and intraspecific variation in coalition formation has been related to extrinsic factors, for instance the group's demography, the number of males within a group, the availability of suitable partners, as well as intrinsic factors, including diverging fighting abilities, profitability, and coordination costs (Henzi et al., 1999; Alberts et al., 2003; Pandit and van Schaik, 2003; Schülke et al., 2010; Bissonnette et al., 2014). In this paper, we focus on age as an intrinsic factor to explain intraspecific variation in male coalition formation in one semi-free-ranging population of Barbary macaques (*Macaca sylvanus*). It has previously been shown that age can act as a good proxy for the physical condition (Bissonnette et al., 2009a). Older males may be forced to start behaving cooperatively as they age to maintain their access to females (Bissonnette et al., 2009b). Older (post-prime) males in Barbary macaques and savannah baboons have been found to compensate for diminishing fighting abilities by supporting each other in coalitions (Noë, 1992; Bissonnette et al., 2009b, 2011). Increasingly shallow paternity distributions are observed with increasing age range among co-resident males (Alberts et al., 2006). When post-prime males cooperate, they can benefit by increasing their reproductive success to the level of a prime male (Kuester et al., 1995). Until now, we lacked the longitudinal data to answer the question of whether a male's reproductive strategy changes with age. In the current study, we took age as a proxy for the male's intrinsic power (Berghänel et al., 2011) and investigated whether it is an important factor influencing coalition formation in Barbary macaque males using a combined cross-sectional and longitudinal approach comparing three study years (2006, 2008, and 2014). Inspection of the relationship between age and competitive ability (Bissonnette et al., 2009a) suggested a steady decrease after age 14 (see below). Thus, we predicted first that post-prime phase males (≥ 14 years) form coalitions more often than prime males (5–13) in the same group year (in 2006, 2008, or 2014 respectively). Second, we predicted that the five males that changed from prime to post-prime age between 2008 and 2014 would switch from a solo strategy to a cooperative competitive strategy and thus increase their coalitionary activity.

## 2 Methods

### 2.1 Ethics statement

This study complies with the German regulations in terms of ethical treatment of research subjects. Affenberg Salem is a private facility. The scientific directors of the enclosure gave permission to conduct the study. This study was exclusively non-invasive and based on observational data collection. No subject experienced any impact on its welfare. Barbary macaques have been classified as an endangered species by the IUCN (2016).

### 2.2 Study site

**Figure 1:**
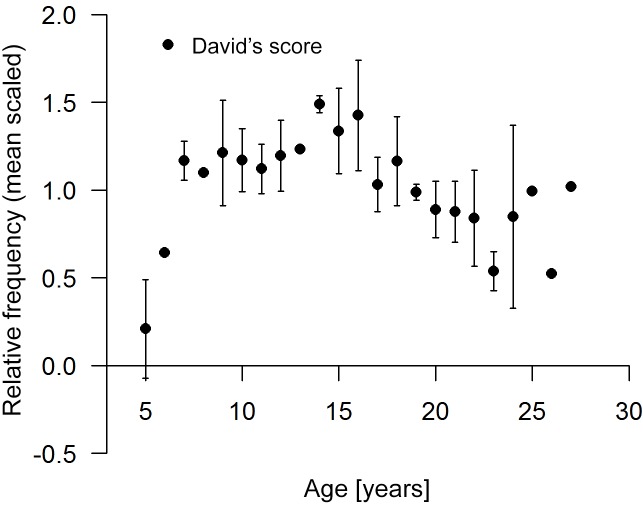
Relative David's scores ± standard deviation distributed across age. All values were corrected for each period by mean scaling (divided by the mean of the respective period).

We conducted the study at Affenberg Salem (Germany). The 20 ha enclosure comprised mixed woodland and held 200 Barbary macaques organized in three freely interacting social groups consisting of 50 to 80 individuals living under semi-free-ranging conditions (R. Hilgartner, personal communication, 2014; de Turckheim and Merz, 1984). Study group H was fed daily in the morning (around 08:30) with various vegetables, fruits, and grains. Monkeys also fed on insects as well as natural vegetation like beechnuts, grass, roots, and leaves and had ad libitum access to water and monkey chow. To investigate the age dependency in coalition formation, we combined data collected on the same group in 2006 (Bissonnette et al., 2009b), 2008 (Berghänel et al., 2011), and the current study period 2014 (Table 1). In the three study periods the group comprised a minimum of 18 males (age range from 5 to 29 years). For detailed information on group composition see Table 1. For each data collection year, males were assigned either to the prime (5–13 years old) or to the post-prime (≥ 14 years old) category following Bissonnette et al. (2009b) and our own investigation of the relationship between competitive ability (normalized David's scores, de Vries et al., 2006) and age across all three study years (Fig. 1). All our results are robust against changing this cut-off to 15 or 16 years of age. We were able to clearly identify all individuals by their physical appearance in terms of face shape and pigmentation, posture, scars, injuries, and missing fingers or toes. We could identify females reliably from their sexual swelling's size, shape, and colour. Exact age was known from demographic records provided by the park management (R. Hilgartner, personal communication, 2014).

**Table 1:** Male ID, age in years, age class (juvenile, Juv: ≤ 2 years old, subadult, Sub: ≤ 4 years old, prime: 5–13 years old, post-prime: ≥ 14 years old) of all males in the study group 2006, 2008, and 2014. Bold font indicates males that changed their age class from prime to post-prime from 2008 to 2014.

	2006		2008		2014	
Male ID	Age	Age class	Age	Age class	Age	Age class
Y2	25	Post-prime	27	Post-prime		
Z30	24	Post-prime	26	Post-prime		
C5	21	Post-prime	23	Post-prime		
C13	21	Post-prime	23	Post-prime		
D10	20	Post-prime	22	Post-prime		
D11	20	Post-prime	22	Post-prime		
D13	20	Post-prime	22	Post-prime		
D25	20	Post-prime	22	Post-prime		
D27	20	Post-prime				
D29	20	Post-prime	22	Post-prime		
E13	19	Post-prime	21	Post-prime		
E14	19	Post-prime	21	Post-prime		
F3	18	Post-prime	20	Post-prime		
G4	17	Post-prime	19	Post-prime		
H3	16	Post-prime	18	Post-prime	24	Post-prime
I2	15	Post-prime	17	Post-prime	23	Post-prime
J3	14	Post-prime	16	Post-prime	22	Post-prime
L1	12	Prime	14	Post-prime	20	Post-prime
**M1**	11	Prime	**13**	**Prime**	**19**	**Post-prime**
**N1**	10	Prime	**12**	**Prime**	**18**	**Post-prime**
**N2**	10	Prime	**12**	**Prime**	**18**	**Post-prime**
**O2**	9	Prime	**11**	**Prime**	**17**	**Post-prime**
O3	9	Prime	11	Prime		
**Q7**	7	Prime	**9**	**Prime**	**15**	**Post-prime**
U1	3	Sub	5	Prime${}^{*}$	11	Prime
W1	2	Sub	4	Sub	10	Prime
W4	2	Sub	4	Sub	10	Prime
Y3			2	Sub	8	Prime
Z1			1	Juv	7	Prime
Z3			1	Juv	7	Prime
A1			0	Juv	6	Prime
B3					5	Prime
B4					5	Prime

${}^{*}$ no data available.

### 2.3 Behavioural data collection

We collected 279, 906, and 590 h of male focal animal data using continuous recording during the mating seasons (October until February) in 2006, 2008, and 2014 respectively (Altmann, 1974; Martin and Bateson, 1993). Males were followed using a 15 min (2006) and 45 min protocol (2008, 2014). We collected data on coalitionary aggression ad libitum throughout all the study periods (Martin and Bateson, 1993). Coalition formation is a specific form of cooperative behaviour which we define here as two individuals showing joint aggression against a common target (Bercovitch, 1988). We used an inclusive definition including coalitions that occur as parallel coalitions when two individuals simultaneously initiate aggression towards a third party (Noë, 1994) or as interference in an already ongoing fight (Noë, 1994; Silk, 1992). During ad libitum observations we often missed the beginning of a polyadic conflict and were not able to classify coalitions as parallel or interference coalitions. We excluded coalitions of more than two partners as well as scream fights due to difficulties in analysis. For coalitions, we collected data on date and time, target identity, and coalition partner identity. If possible, we recorded the initial conflict situation that led to the coalition formation, submission, or counter-aggression of the target and whether one partner abandoned the other during the coalition. Males left a coalition in only eight cases (three times a prime male left, five times a post-prime male). We recorded coalitions using Handheld Kodak Zx1 HD and Panasonic Lumix DMC-TZ36 cameras. Videos provided a spoken commentary on male IDs and spatial position in the ongoing conflict for improvement of recognition of individuals during later video analysis.

### 2.4 Statistical analysis

For each male, we included his absolute frequency of participation in coalitions into the behavioural analysis. These counts needed no adjustment for observation time because all males were observed equally long. For comparison between years we used relative frequencies. We used permutation tests to assess whether the observed coalition pattern regarding the two age classes (prime and post-prime) differed from a random pattern. The permutation tests randomly shuffled observed values keeping the original number of observations and nodes constant. The test statistic was recalculated for every permutation cycle. We used the exact Mann–Whitney U test implemented in the *coin* package (Hothorn et al., 2008) by R 3.1.2 (R Core Team, 2014) to analyse differences between age classes in participation in coalitions as partners in each study year. To assess changes in coalition formation from 2008 to 2014 in those males that age from prime to post-prime between these years we used paired Wilcoxon tests. Since the total number of coalitions observed differed between years (150 in 2008 and 71 in 2014), we used relative frequencies to compare the two data sets. We calculated relative frequencies dividing the absolute frequency of participation by the overall number of coalitions in the respective year. All graphics were visualized by making use of R 3.1.2 (R Core Team, 2014). Directed predictions were derived a priori from theory and published literature. Consequently, one-tailed tests were used throughout.

## 3 Results

**Figure 2:**
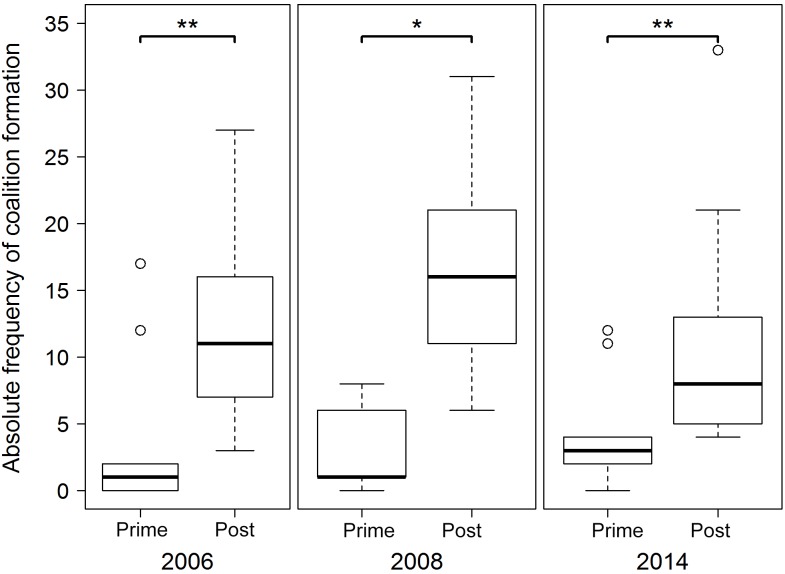
Cross-sectional analysis of coalition formation (absolute frequencies). Asterisks denote significance level at P < 0.05 (${}^{*}$) and < 0.01 (${}^{**}$) in one-tailed exact Mann–Whitney U tests comparing prime and post-prime coalition formation.

During all study years, the majority of coalitions were formed by post-prime males joining each other (67 % in 2006, 52 % in 2008, 39 % in 2014). In cross-sectional analyses age classes differed in their coalitionary activity. The participation in coalitions was significantly higher in post-prime males than in prime males in every study year with 3- to 10-fold increases (difference between medians) in absolute frequencies of coalitions (exact Mann–Whitney U test: 2006: Z=2.811, N=24, P < 0.01; 2008: Z=3.438, N=23, P < 0.05; 2014: Z=2.479, N=18, P < 0.01; Fig. 2).

**Figure 3:**
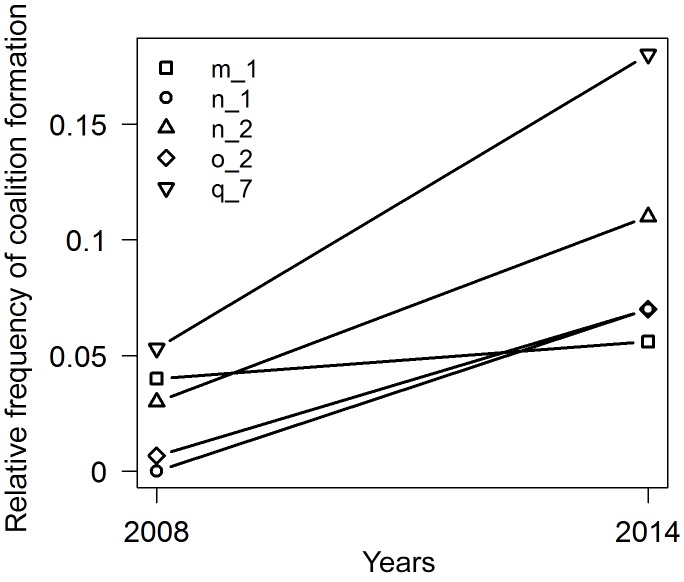
Relative frequency of coalition formation of five individuals changing from prime (2008) to post-prime phase (2014).

In accordance with our prediction the longitudinal analysis showed that males that had aged into the post-prime phase formed 2.36 times more (median of increases) coalitions in 2014 than in 2008 (paired Wilcoxon test, V=0, N=5, P < 0.05, Fig. 3). To assess whether the aged males increased their coalitionary activity to a level comparable to other older males, coalition formation of these five males was compared to the coalition formation by all post-prime males in 2008 (Mann–Whitney U test, W=43, N=21, P=0.66) and no difference was detected.

## 4 Discussion

This study aimed at investigating whether a male's propensity to form within-group coalitions is driven by a change in competitive strategy depending on his age class. Reaching a certain age has previously been proposed to affect the within-group distribution of coalitions in male Barbary macaques (Bissonnette et al., 2009b). When growing older, competitive ability diminishes and males engage in alternative strategies to optimize their reproductive success (Noë, 1994; Bissonnette et al., 2009b). In accordance with this prediction, in all three study years post-prime males formed more coalitions than prime males. The group had roughly equal proportions of prime and post-prime males in all years. Yet, 13 post-prime males present in 2006 died and 5 males matured into their prime before 2014, leaving only 9 males that were present in all study years. Therefore, we consider the three analyses as biologically rather independent. As the crucial advancement over past studies our longitudinal analysis showed that all five males that changed age classes between 2008 and 2014 increased their frequency of coalition formation supporting the prediction that coalition formation is condition dependent (Bissonnette et al., 2011). The number of post-prime males in the study group is rather large compared to groups of Barbary macaques in the wild (Young et al., 2013). Yet, observations on baboons suggest that similar age-dependent strategies pertain in the wild with older males being more engaged in coalition formation than younger males, potentially to dampen the negative consequences of an age-related drop in the social status leading to a decrease of the reproductive success (Alberts et al., 2003, 2006).

Support for age-related intraspecific variation in coalition formation comes from several studies of nonhuman primates and a few other mammalian species where older males, mostly but not necessarily low-ranking, successfully formed coalitions against young, mostly higher-ranking males (Bercovitch, 1988; Noë and Sluijter, 1995; Mitani et al., 2002; de Villiers et al., 2003; Berghänel et al., 2010; Bissonnette et al., 2011). Age can also affect coalitionary patterns in different ways. For example, in wild dogs (*Lycaon pictus*), males from the same age class support each other in conflicts more often than males from different age classes (de Villiers et al., 2003). In Atlantic spotted dolphins (*Stenella frontalis*), coalition strength increases with aging into the next age class (Green et al., 2011). Age has been included as a contributing factor but was rarely in the focus of these studies, neglecting its importance in shaping the reproductive strategy a male is pursuing during its lifetime.

Male alternative reproductive strategies act on different levels in the competition over reproductive success. Coalition can have a direct effect on mating success by preventing others from mating and lead to a starting point for a consort with the respective female (Bercovitch, 1988; Noë and Sluijter, 1990; Bissonnette et al., 2011). Just the threat of being the target of a coalition by signalling recruitment to another male has been found to lead to a changeover in consort male identity (Bissonnette et al., 2011). Yet, coalitions do not always happen in a sexual context where consort changeover leads to direct access to females (Berghänel et al., 2010; Young et al., 2014b; Bissonnette et al., 2015). Without female presence coalitions can still indirectly affect variation of mating success in a group and thus can be seen as a pre-mating strategy to improve future success by intimidation of opponents (Berghänel et al., 2010). It can also have an effect on dyadic dominance relationships, which further leads to rank changes and thus facilitates access to females (Berghänel et al., 2011; Young et al., 2014a; Ostner and Schülke, 2014). We predicted that males would switch from a solo strategy to a cooperative competitive strategy once they passed their prime age and thus peak physical ability. Several studies on macaques and baboons showed that post-prime males with diminished competitive ability are still able to win a conflict against a more dominant younger male if they cooperate with each other (Noë, 1992; Alberts et al., 2003; Bissonnette et al., 2009b, 2011). Younger prime males, in contrast, are better equipped to defend females and pursue a solo strategy (Smuts and Watanabe, 1990). By competing on their own, young males are expected to profit from exclusive mating opportunities (Noë and Sluijter, 1990; Smuts and Watanabe, 1990). The role coalitions play as a pre-mating strategy should be addressed in future empirical studies to understand how and to what extent coalition formation can level within-group lifetime reproductive success. In general, studies focusing on age in a competitive context are scarce. Especially in non-primate species, difficulties arise in determining the individual age in a natural field setting, calling for precise morphological and behavioural criteria (Bercovitch, 1988; Mitani et al., 2002). For future studies on coalitionary activity our results illustrate the importance of age as an intervening variable in cooperative competition and the importance of considering life history and changing reproductive strategies as factors determining coalition formation patterns.

## 5 Data availability

The data used in this study are available in the Supplement.

## Supplement

10.5194/pb-4-1-2017-supplement The supplement related to this article is available online at: https://doi.org/10.5194/pb-4-1-2017-supplement.
