# Supplementary material for: Age-dependent change of coalitionary strategy in male Barbary macaques
Source: Primate Biol. 2017 Jan 26;4(1):1–7. doi: 10.5194/pb-4-1-2017 (PMC7041515; doi:10.5194/pb-4-1-2017)
Supplement: The supplement related to this article is available online at: https://doi.org/10.5194/pb-4-1-2017-supplement. [file pb-4-1-supplement.pdf]

Supplement of Primate Biol., 4, 1–7, 2017
http://www.primate-biol.net/4/1/2017/
doi:10.5194/pb-4-1-2017-supplement
© Author(s) 2017. CC Attribution 3.0 License.

Supplement of

Age-dependent change of coalitionary strategy in male Barbary macaques

Eva-Maria Rathke et al.

Correspondence to: Eva-Maria Rathke (erathke@dpz.eu)

The copyright of individual parts of the supplement might differ from the CC-BY 3.0 licence.

data_year	ID	coalitions
2006	c_13	27
2006	c_5	4
2006	d_10	8
2006	d_11	5
2006	d_13	15
2006	d_25	14
2006	d_27	18
2006	d_29	15
2006	e_13	8
2006	e_14	11
2006	f_3	16
2006	g_4	17
2006	h_3	9
2006	i_2	3
2006	j_3	7
2006	l_1	1
2006	m_1	1
2006	n_1	0
2006	n_2	0
2006	o_2	2
2006	o_3	0
2006	q_7	0
2006	y_2	17
2006	z_30	12
2008	y_2	13
2008	z_30	12
2008	c_5	16
2008	c_13	21
2008	d_10	11
2008	d_11	9
2008	d_13	6
2008	d_25	16
2008	d_29	30
2008	e_13	16
2008	e_14	21
2008	f_3	19
2008	g_4	15
2008	h_3	31
2008	i_2	10
2008	j_3	25
2008	l_1	8
2008	m_1	6
2008	n_1	0
2008	n_2	5
2008	o_2	1
2008	o_3	1
2008	q_7	8
2014	h_3	21
2014	i_2	7

2014	j_3	33
2014	l_1	8
2014	m_1	4
2014	n_1	5
2014	n_2	8
2014	o_2	5
2014	q_7	13
2014	u_1	2
2014	w_1	0
2014	w_4	1
2014	y_3	3
2014	z_1	4
2014	z_3	12
2014	a_1	11
2014	b_3	2
2014	b_4	3
